# Parent-Reported Assessment Scores Reflect the ASD Severity Level in 2- to 7-Year-Old Children

**DOI:** 10.3390/children9050701

**Published:** 2022-05-10

**Authors:** Priyanka Jagadeesan, Adam Kabbani, Andrey Vyshedskiy

**Affiliations:** 1 Boston University, Boston, MA 02215, USA; priyanka.jagadeesan20@imperial.ac.uk (P.J.); akabbani@bu.edu (A.K.); 2 ImagiRation, Boston, MA 02135, USA

**Keywords:** autism, ASD, language delay, receptive language, language comprehension, combinatorial language, recursive language, prefrontal synthesis, syntactic language

## Abstract

We investigated the relationship between parent-reported assessments and autism spectrum disorder (ASD) severity level. Parents evaluated 9573 children with ASD on five subscales—combinatorial receptive language, expressive language, sociability, sensory awareness, and health—using the Autism Treatment Evaluation Checklist (ATEC) and Mental Synthesis Evaluation Checklist (MSEC). The scores in every subscale improved with age, and there were clear differences between the three diagnostic categories. The differences between mild and moderate ASD, and moderate and severe ASD reached statistical significance in each subscale and in every age group in children 3 years of age and older. These findings demonstrate a consistent relationship between children’s diagnoses and their assessments and provide evidence in support of the reliability of parent-report evaluations for ASD. Additionally, this is the first investigation of the relationship between ASD severity level and the ATEC/MSEC scores for the age range from 2 to 7 years.

## 1. Introduction

Clinical trials routinely use parent-reported assessments of children as an outcome measure [1,2,3,4]. Parents’ assessments provide additional insights into the course of a disease without imposing the extra cost associated with clinicians’ assessments. However, little data exist on the reliability of parent-report evaluations [5]. In 2015, we published a language training app for children [6,7,8,9,10] inviting parents to evaluate their child’s development every three months. The parents completed a Autism Treatment Evaluation Checklist (ATEC) [11] and a Mental Synthesis Evaluation Checklist (MSEC) [12] that assess children along five subscales: combinatorial receptive language, expressive language, sociability, sensory awareness, and health. Resultantly, more than 100,000 assessments were gathered.

The analysis of these parents’ assessments yielded several important insights into the effects of culture and physical conditions on the developmental trajectories of children with ASD. A longitudinal study investigating the impact of passive video and television watching in children with ASD (N = 3227) demonstrated that greater exposure to video and television watching was correlated with a 1.3-fold (*p* = 0.0719) faster improvement in the development of expressive language, but also resulted in a 1.4-fold (*p* = 0.0128) slower development of combinatorial receptive language. The differences in the sociability, sensory awareness, and health scores remained insignificant [13]. Similarly, a prospective 3-year study looking at pretend play (N = 7069) showed that pretend play was associated with a 1.9-fold faster improvement in combinatorial receptive language (*p* < 0.0001), a 1.4-fold faster improvement in expressive language (*p* < 0.0001), and a 1.3-fold faster improvement in sensory awareness (*p* = 0.0009); meanwhile, the effects on sociability and health were insignificant. In terms of health studies, seizures and sleep have been analyzed for their impact on development in children with ASD. An analysis of the effect of seizures (N = 8461) showed that children with no seizures improved their expressive language 1.3-times faster (*p =* 0.0037), their sociability 2.3-times faster (*p =* 0.0320), their sensory awareness 6.2-times faster (*p* = 0.0047), and their health 20.0-times faster (*p* < 0.0001), whereas the effect on receptive language was insignificant [14]. Additionally, an investigation of the effect of sleep problems (N = 7069) showed that children with no sleep problems improved their sociability 3-times faster (*p* = 0.0426) and their health significantly faster (*p* < 0.0001; the exact ratio could not be calculated as the health score in children with sleep difficulties had declined relative to the baseline); the effects on receptive language, expressive language, and sensory awareness were insignificant [15]. Finally, in a 3-year study of 6454 children, those who engaged with a specialized language therapy improved their combinatorial language scores 2.2-times faster compared to children with comparable initial evaluations (*p* < 0.0001) and improved their expressive language score 1.4-times faster (*p* = 0.0144). However, the differences in their sociability, sensory awareness, and health scores remained insignificant [16].

Though these results provide interesting correlations for the impacts of multifactorial cultural and physiological conditions on ASD development, there remains resistance amongst researchers in accepting parent-reported evaluations. There is a common belief within the psychological community that parents can yield to wishful thinking, and therefore may not be reliable when assessing their own children [17]. In order to provide clarity on the reliability of parent reports, we investigated the relationship between children’s evaluation scores and their ASD severity. The Diagnostic and Statistical Manual of Mental Disorders, 5th Edition (DSM-5), specifies three levels of ASD, depending on severity of the disorder and the support required in daily life [18]. We hypothesized that if parents clearly understood and honestly communicated their child’s diagnosis, the reported ASD severity level would have a consistent relationship with the assessment subscales. Greater ASD severity would correspond to worse assessment scores, and vice versa. Conversely, if parents misreported their children’s diagnosis, no difference in the average assessment score would be expected between the groups.

The cross-sectional analysis of 9573 children has demonstrated statistically significant differences between levels of ASD, i.e., between mild and moderate, and moderate and severe ASD diagnosis. These differences were seen within each subscale in every age group of 3 years and older. These findings are consistent with the high reliability of parent-reported evaluations and their children’s diagnoses.

## 2. Methods

### 2.1. Participants

The participants consisted of 9573 users of a language therapy application, which was made available free of charge in app stores in September 2015. Upon downloading the app, the caregivers were asked to register to input demographic details, including the child’s diagnosis and age. The caregivers were asked to select a single diagnosis from the following list: Suspected Autism, Mild Autism, Moderate Autism, Severe Autism, Pervasive Developmental Disorder, Lost Diagnosis of Autism or PDD, Asperger Syndrome, Social Communication Disorder, Specific Language Impairment, Apraxia, Sensory Processing Disorder, Down Syndrome, Other Genetic Disorder, Mild Language Delay, ADHD or ADD, and Normally Developed Child. This study included participants with Mild Autism, Moderate Autism, Severe Autism, or Asperger Syndrome. The Asperger Syndrome group was combined with the Mild Autism group, as per DSM-5 recommendations. Table 1 describes the group size and gender information.

The education level of the participants’ caregivers was as follows: 94% had at least a high school diploma, 73% had at least college education, 35% had at least a master’s degree, and 5% had a doctorate. All of the data presented in this report are cross-sectional.

### 2.2. Assessments

The users agreed to anonymized data analysis and completed the Mental Synthesis Evaluation Checklist (MSEC) [12] to evaluate combinatorial receptive language, and the Autism Treatment Evaluation Checklist (ATEC) [11] to evaluate development on four subscales: (1) Speech/Language/Communication, (2) Sociability, (3) Sensory/Sensory awareness, and (4) Physical/Health/Behavior. The first ATEC subscale—referred to here as Expressive Language—contains 14 items with scores ranging from 0 to 28 points. The Sociability subscale contains 20 items with a score range of 0 to 40 points. The third subscale—referred to here as the Sensory Awareness subscale—has 18 items, and its scores range from 0 to 36 points. The fourth subscale—referred to here as the Health subscale—contains 25 items, and its scores range from 0 to 75 points. The scores from each subscale are combined to calculate a Total Score, which ranges from 0 to 179 points. The scores are positively correlated with ASD severity. Thus, a lower score indicates a lower severity, and a higher score indicates a greater severity of ASD.

### 2.3. Combinatorial Receptive Language Assessment

The MSEC evaluation was designed to be complementary to the ATEC in measuring combinatorial receptive language. Out of the 20 MSEC items, items that directly assess receptive language are as follows: (1) Understands simple stories that are read aloud; (2) Understands elaborate fairy tales that are read aloud (i.e., stories describing fantasy creatures); (3) Understands some simple modifiers (i.e., green apple vs. red apple, or big apple vs. small apple); (4) Understands several modifiers in a sentence (i.e., small green apple); (5) Understands size (can select the largest/smallest object out of a collection of objects); (6) Understands possessive pronouns (i.e., your apple vs. her apple); (7) Understands spatial prepositions (i.e., put the apple on top of the box vs. inside the box vs. behind the box); (8) Understands verb tenses (i.e., “I will eat an apple” vs. “I ate an apple”); (9) Understands the change in meaning when the order of words is changed (i.e., understands the difference between “a cat ate a mouse” vs. “a mouse ate a cat”); (10) Understands explanations about people, objects or situations beyond the immediate surroundings (e.g., “Mom is walking the dog, “The snow has turned to water”). The MSEC is comprised of 20 questions with a score range of 0 to 40 points. Like the ATEC, a lower MSEC score is indicative of more developed combinatorial receptive language. 

The psychometric quality of the MSEC was tested with 3715 parents of ASD children [12]. The MSEC showed good internal reliability (Cronbach’s alpha > 0.9). Additionally, it also displayed adequate test–retest reliability, good construct validity and known-group validity. The MSEC norms are reported in Ref. [19].

In order to simplify the interpretation of the figure labels, subscale 1 of the ATEC evaluation is herein referred to as the Expressive Language subscale, and the MSEC scale is referred to as the Receptive Language subscale.

## 3. Results

**Figure 1:**
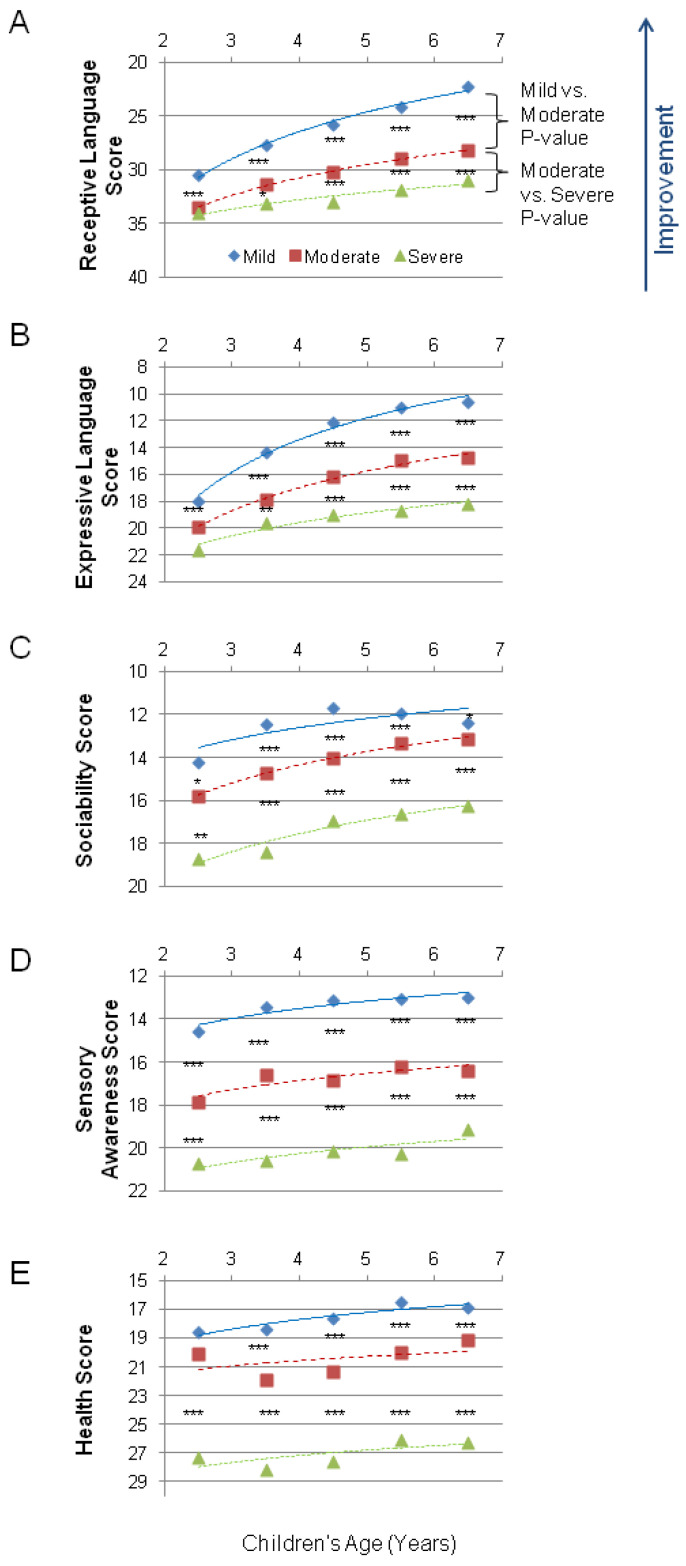
Averaged scores in five subscales for the mild, moderate, and severe ASD groups. A lower score indicates symptom improvement. The *p*-value is marked: *** <0.0001, ** <0.001, and * <0.05. (**A**) Receptive Language score. (**B**) Expressive Language score. (**C**) Sociability score. (**D**) Cognitive awareness score. (**E**) Health score.

The participants were 9573 parents of children with ASD ranging from 2 to 7 years old. They identified their children’s diagnosis as mild, moderate, or severe ASD, and assessed their children on five subscales: combinatorial receptive language, expressive language, sociability, sensory awareness, and health. The mild ASD group showed superior scores compared to the moderate ASD group, and the moderate ASD group had better scores compared to the severe ASD group in all of the subscales across all ages (Figure 1).

On the Receptive Language subscale (Table 2), the difference between the severe ASD group and the moderate ASD group was statistically significant in children of 3 years of age (*p* = 0.008), as well as in the age groups of 4, 5, and 6 years of age (*p* < 0.0001). However, the difference did not reach statistical significance in 2-year-olds (*p* = 0.53). In contrast, the difference between the groups with moderate ASD and mild ASD was statistically significant (*p* < 0.0001) in every age group studied.

The Expressive Language subscale (Table 3) showed a similar trend to the scores for receptive language, with the difference between the severe ASD group and the moderate ASD group being statistically significant in children 3 years of age (*p* = 0.008), as well as in the age groups of 4, 5, and 6 years of age (*p* < 0.0001), but not in the 2 years of age group (*p* = 0.46). The difference between the moderate ASD group and the mild ASD group was statistically significant (*p* < 0.0001) in every age group.

On the Sociability subscale (Table 4), the difference between the severe ASD group and the moderate ASD group was statistically significant in children 2 years of age (*p* = 0.0004) and in the age groups of 3, 4, 5, and 6 years of age (*p* < 0.0001). The difference between the moderate ASD group and the mild ASD group was statistically significant in children 2 years of age (*p* = 0.012), as well as in the groups for 3, 4, 5 (*p* < 0.0001), and 6 years of age (*p* = 0.03).

On the Sensory awareness subscale (Table 5), the differences between the severe ASD group and the moderate ASD group were statistically significant in every age group (*p* < 0.0001). The difference between the moderate ASD group and the mild ASD group was also statistically significant in every age group (*p* < 0.0001).

On the Health subscale (Table 6), the difference between the severe ASD group and the moderate ASD group was statistically significant in children in every age group (*p* < 0.0001). The difference between the moderate ASD group and the mild ASD group had not reached statistical significance in the 2 years of age group (*p* = 0.526), but was statistically significant (*p* < 0.0001) in older children.

**Figure 2:**
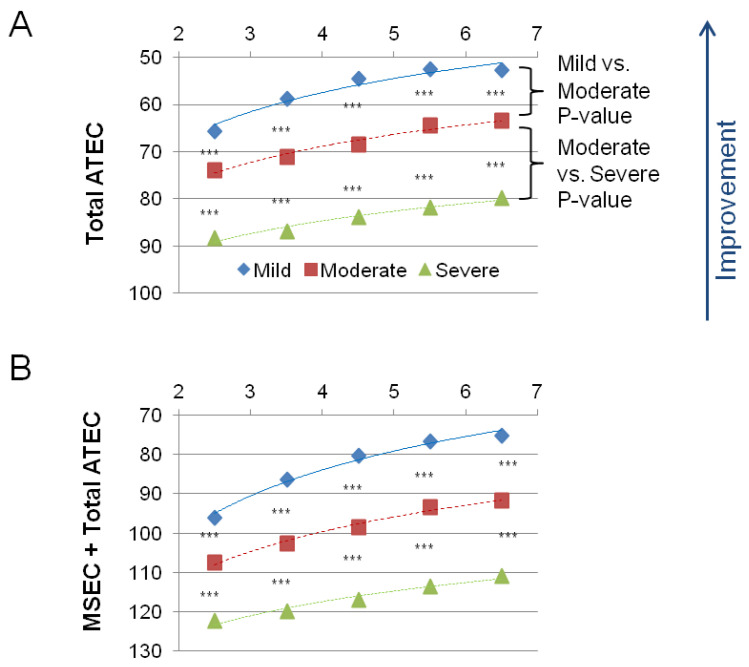
Averaged scores in five subscales for the mild, moderate, and severe ASD groups. Lower scores indicate symptom improvement. (**A**) The total ATEC score, which is the sum of the Expressive Language score, Sociability score, Cognitive awareness score, and Health score. (**B**) The sum of the total ATEC and the MSEC scores (i.e., the sum of all five subscales). The *p*-value is marked: *** <0.0001.

Figure 2 shows the combined measures: the Total ATEC score (Table 7) and the MSEC score + the Total ATEC score (Table 8). In both combined measures, the difference between the severe ASD group and the moderate ASD group, and between the moderate ASD group and the mild ASD group was statistically significant in children in every age group (*p* < 0.0001).

## 4. Discussion

This study evaluates the reliability of parents’ assessments by comparing parent-reported evaluation scores with children’s ASD severity. Despite the common use of parent-report assessments in clinical trials [1,2,3,4], there is little research evaluating their reliability [17]. Because we had access to both parent-reported assessments and children’s diagnoses, we had the opportunity to compare these scores between diagnoses. To our satisfaction, we have found significant differences between the diagnostic categories. As expected, children diagnosed with mild ASD had better scores than children with moderate ASD, and children diagnosed with moderate ASD had better scores than those with severe ASD. The differences reached statistical significance in each subscale and in every age group for children 3 years and older, as well as in the cumulative Total ATEC and Total ATEC + MSEC scales for children 2 years of age and older. These findings suggest the high reliability of parents’ assessments. 

On the combinatorial receptive language subscale, significant differences were detected between mild and moderate ASD at 2 years of age (*p* < 0.0001, Figure 1A). This observation combined with the observation of a significant difference between MSEC scores when comparing neurotypical and ASD children by Arnold et al. [19] suggests that the assessment of combinatorial receptive language holds merit for the diagnosis and monitoring of language deficits.

Consistent with previous reports [20], the difference in combinatorial receptive and expressive language scores between ASD levels increased with age, with mild ASD showing greater gains compared to moderate and severe ASD (Figure 1A,B). It is crucial to note that even mild ASD scores remain significantly behind the normal range (compare a mild ASD combinatorial receptive language score of 30.4 ± 7.5 at 2 years of age to the score of 22.8 ± 4.2 observed in neurotypical children at the same age; at 6 years of age: the score is 22.3 ± 9.2 versus 4.5 ± 2.4) [19]. Conversely, the difference in the sociability, sensory awareness, and health scores between the ASD levels decreased or remained unchanged with age (Figure 1C–E).

Additionally, the reported data improve the interpretation of ATEC and MSEC scores. ATEC and MSEC are freely available online (www.autism.org and www.imagiration.com, accessed 8 May 2022), have been translated into multiple languages, and are used by tens of thousands of families annually to monitor their children’s ASD symptoms. It is important to note that ATEC and MSEC were designed to assess the effectiveness of treatment, and are not diagnostic checklists [11,12]. Therefore, ASD severity can only be approximated by the total ATEC/MSEC scores and age. Table 9 and Table 10 list the approximated ATEC total scores and approximated MSEC + ATEC total scores as they relate to ASD severity level and age, respectively.

Note that unlike the standardized Autism Diagnostic Observation Schedule (ADOS) [21] and the Childhood Autism Rating Scale (CARS) [22], the ATEC and MSEC diagnostic cutoffs are age-dependent. ADOS achieves diagnostic cutoff age-independence by normalizing the raw score differently for each age range. CARS achieves diagnostic cutoff age-independence by assessing abilities against the expected developmental growth of a typical child. ATEC and MSEC provide a raw score that is not normalized for each age. Furthermore, all ATEC and MSEC items only assess ASD signs and symptoms, and never require a comparison to a typically developing child, as parents may not be familiar with the expected developmental growth of a typical child.

## 5. Limitations

The data for this study were provided by 9573 users of a free language therapy app. There is a concern in the autism community that an app could take time away from joint engagement [23]. We agree that this problem exists for addictive apps such as YouTube. Our language therapy app, on the other hand, is completely non-addictive. It requires constant focus and concentration, which is difficult for children to maintain for a long time. As a result, children can only play with the app for a short period of time. The average app-use is 2 days/week; on the days when the app is used, the average use duration is 15 min [7]. Most of the time (58%), parents work together with their children on the app exercises. Furthermore, MITA teaches language therapy techniques to parents, and encourages them to use these techniques outside of the app. Parents are trained to continue playing language therapy games in the kitchen, on the playground, on the beach, and anywhere else they go with their child. For example, in the kitchen, parents are encouraged to ask their child to “put the cup {on | under | in front of | behind} the plate.” Similarly, on the playground, parents are taught to find a white wooden chip and a black wooden chip, and to ask their child to “put the white chip {on | under | in front of | behind} the black chip.” On the beach, parents are instructed to find a white pebble and a black pebble and ask their child to “put the white pebble {on | under | in front of | behind} the black pebble.” When their child is ready, parents are encouraged to make the game more complex by adding color, size and number modifiers. For example, “put two red pencils {on | under | in front of | behind} the plate.” The parents also learn to give instructions using different word orders in order to avoid routinization. In an online survey of the app users, more than half of the responders reported that they had learned language therapy techniques from the app exercises.

Epidemiological studies of app users provide access to a large number of children at a relatively low cost, but have obvious downsides, such as relying on parent reports for diagnosis and assessments. In fact, parents may yield to wishful thinking and overestimate their children’s abilities [17]. Future studies should compare parent-report instruments such as ATEC and MSEC to clinicians’ assessments, such as CARS and ADOS, in order to further explore the reliability of parents’ evaluations.

This study contributes to the affirmation of the consistency of parents’ assessments in the evaluation of their children with ASD, and provides additional evidence in support of reliability of such evaluations for autism spectrum disorder [24]. To our knowledge, this is the first investigation of the relationship between parent-report ATEC/MSEC scores and ASD severity for the age range of 2- to 7-year-old children.

## Figures and Tables

**Table 1:** The number of participants in each age group and ASD level. The number in brackets shows the percentage of males.

	Age (Years)				
	2	3	4	5	6
**Mild ASD**	295 (73%)	868 (78%)	1076 (84%)	882 (80%)	587 (79%)
**Moderate ASD**	242 (74%)	914 (78%)	1136 (76%)	1158 (77%)	1077 (81%)
**Severe ASD**	82 (63%)	186 (78%)	314 (79%)	332 (80%)	424 (80%)

**Table 2:** Averaged Combinatorial Receptive Language score (assessed by MSEC) for the mild, moderate, and severe ASD groups. A lower score indicates symptom improvement. The data are presented as the mean (SD).

	Age (Years)				
	2	3	4	5	6
**Mild ASD**	30.4 (7.5)	27.7 (7.9)	25.8 (7.7)	24.1 (8.4)	22.3 (9.2)
**Moderate ASD**	33.5 (5.9)	31.4 (6.8)	30.1 (7.3)	28.9 (7.5)	28.2 (7.8)
**Severe ASD**	34 (8)	33.1 (8)	33 (6.8)	31.9 (7.5)	31 (7.9)

**Table 3:** Averaged Expressive Language (assessed by ATEC subscale 1) score for the mild, moderate, and severe ASD groups. A lower score indicates symptom improvement. The data are presented as the mean (SD).

	Age (Years)				
	2	3	4	5	6
**Mild ASD**	18 (5.9)	14.4 (6.7)	12.1 (6.5)	11 (6.2)	10.6 (6.2)
**Moderate ASD**	20 (5.2)	17.8 (5.8)	16.2 (6.2)	14.9 (6.1)	14.7 (5.7)
**Severe ASD**	21.6 (5.3)	19.6 (5.6)	19 (5.6)	18.7 (5.6)	18.1 (5.7)

**Table 4:** Averaged Sociability (assessed by ATEC subscale 2) score for the mild, moderate, and severe ASD groups. A lower score indicates symptom improvement. The data are presented as the mean (SD).

	Age (Years)				
	2	3	4	5	6
**Mild ASD**	14.2 (7.3)	12.4 (7.5)	11.7 (7.3)	11.9 (7.5)	12.3 (7.3)
**Moderate ASD**	15.8 (7.3)	14.7 (7.5)	14 (7.2)	13.3 (6.9)	13.1 (6.9)
**Severe ASD**	18.7 (7.9)	18.4 (7.7)	16.9 (7.8)	16.6 (8.1)	16.3 (7.7)

**Table 5:** Averaged Sensory Awareness (assessed by ATEC subscale 3) score for the mild, moderate, and severe ASD groups. A lower score indicates symptom improvement. The data are presented as the mean (SD).

	Age (Years)				
	2	3	4	5	6
**Mild ASD**	14.6 (6.1)	13.4 (6.5)	13.1 (6.4)	13.1 (6.8)	13 (6.5)
**Moderate ASD**	17.9 (5.8)	16.6 (6.4)	16.8 (6.6)	16.2 (6.1)	16.4 (6.3)
**Severe ASD**	20.7 (6.4)	20.6 (6.7)	20.2 (6.2)	20.3 (6.2)	19.1 (6.5)

**Table 6:** Averaged Health (assessed by ATEC subscale 4) score for the mild, moderate, and severe ASD groups. A lower score indicates symptom improvement. The data are presented as the mean (SD).

	Age (Years)				
	2	3	4	5	6
**Mild ASD**	18.6 (11.8)	18.4 (7.9)	17.6 (11.3)	16.5 (10.9)	16.9 (10.5)
**Moderate ASD**	20.1 (11.8)	21.9 (12.4)	21.4 (12.2)	19.9 (11.2)	19.2 (10.5)
**Severe ASD**	27.3 (15.1)	28.2 (13.9)	27.6 (12.7)	26 (12.8)	26.3 (13.5)

**Table 7:** Averaged Total ATEC score for the mild, moderate, and severe ASD groups. A lower score indicates symptom improvement. The data are presented as the mean (SD).

	Age (Years)				
	2	3	4	5	6
**Mild ASD**	65.6 (21.3)	58.7 (23.3)	54.5 (23.1)	52.5 (23)	52.8 (22.4)
**Moderate ASD**	73.9 (20.6)	71.1 (23.5)	68.4 (24.3)	64.5 (21.9)	63.4 (21.2)
**Severe ASD**	88.4 (23.9)	86.9 (25.6)	83.9 (23.4)	81.8 (24.1)	79.8 (23.7)

**Table 8:** Averaged Total ATEC + MSEC score for the mild, moderate, and severe ASD groups. A lower score indicates symptom improvement. The data are presented as the mean (SD).

	Age (Years)				
	2	3	4	5	6
**Mild ASD**	96 (23.6)	86.4 (27.9)	80.3 (27.2)	76.6 (27.7)	75.1 (27.7)
**Moderate ASD**	107.4 (23)	102.5 (26.6)	98.6 (28.2)	93.4 (26.1)	91.7 (25.6)
**Severe ASD**	122.4 (25.2)	120 (28.8)	116.9 (25.8)	113.6 (26.6)	110.8 (27.5)

**Table 9:** Approximate relationship between the ATEC total score, age, and ASD severity. At any age, a greater ATEC score indicates greater ASD severity.

	Age (Years)				
	2	3	4	5	6
**Mild ASD**	<70	<65	<61	<58	<58
**Moderate ASD**	70–81	65–79	61–76	58–73	58–72
**Severe ASD**	81–179	79–179	76–179	73–179	72–179

**Table 10:** Approximate relationship between MSEC + ATEC total scores, age, and ASD severity. At any age, a greater score indicates greater ASD severity.

	Age (Years)				
	2	3	4	5	6
**Mild ASD**	<102	<94	<89	<85	<83
**Moderate ASD**	102–115	94–111	89–108	85–104	83–101
**Severe ASD**	115–209	111–209	108–209	104–209	101–209

## Data Availability

De-identified raw data from this manuscript are available from the corresponding author upon reasonable request.
